# C-857-01. Artificial Intelligence and Algorithmic Bias in Burn Care: A Literature Review of Ethical Challenges

**DOI:** 10.1093/jbcr/irag033.101

**Published:** 2026-04-06

**Authors:** Joshua Khorsandi, Jason Mirharooni, Liahm Blank, Abu-Bakr Ahmed, Samir Alkhouri

**Affiliations:** Kirk Kerkorian School of Medicine at University of Nevada, Las Vegas, NV; Florida International University Herbert Wertheim College of Medicine, Miami, FL; Kirk Kerkorian School of Medicine at University of Nevada, Las Vegas, NV; Kirk Kerkorian School of Medicine at University of Nevada, Las Vegas, NV; Kirk Kerkorian School of Medicine at University of Nevada, Las Vegas, NV

## Abstract

**Introduction:**

Artificial intelligence (AI) is increasingly being explored in burn care, with applications in triage, pain management, prognostic scoring, and resource allocation. While these tools offer potential improvements in efficiency and precision, they also raise ethical concerns regarding transparency, accountability, and bias. Current literature on burn care technology largely emphasizes feasibility and outcomes, yet few studies address algorithmic fairness.

**Methods:**

We conducted a structured literature review of peer-reviewed articles published between 2010 and 2024 that examined AI, predictive modeling, or digital tools in burn care and related fields. Databases included PubMed, Embase, and Scopus. Articles were screened for relevance to ethical considerations such as bias, equity, and interpretability. Comparative evidence from broader medical specialties was included to contextualize risks of algorithmic misclassification in vulnerable populations.

**Results:**

Across medical domains, predictive models were found to disproportionately misclassify outcomes for racial and ethnic minorities, patients with socioeconomic disadvantage, and individuals with comorbidities. Few burn-specific studies explicitly measured these disparities. The literature highlights insufficient attention to dataset diversity, limited transparency in model development, and lack of systematic auditing for fairness. Ethical discussions remain fragmented, and burn care research lags behind other specialties in interrogating these issues.

**Conclusions:**

AI has potential to enhance burn care delivery but carries risks of amplifying health disparities if fairness safeguards are not prioritized. A paradigm shift is needed from technical validation alone toward embedding ethical frameworks into research and deployment.

**Applicability to Practice:**

Clinicians and researchers must advocate for representative datasets, independent auditing, and mechanisms for accountability in AI-assisted burn care. Integrating these safeguards will promote equity, ensure responsible clinical adoption, and protect vulnerable patient populations from unintended harm.

